# Medical student exposure to women’s health concepts and practices: a content analysis of curriculum at Canadian medical schools

**DOI:** 10.1186/s12909-021-02873-8

**Published:** 2021-08-18

**Authors:** Natalie N. Anderson, Anna R. Gagliardi

**Affiliations:** Toronto General Hospital Research Institute, University Health Network, 200 Elizabeth Street. 13EN-228, M5G2C4 Toronto, Canada

**Keywords:** Women’s health, Health care inequities, Physician training, Medical curriculum, Content analysis

## Abstract

**Background:**

Women’s health (WH) includes a broad array of concerns and challenges that affect health across the lifespan. Considerable research shows that women continue to experience disparities in access to and quality of care. Apart from surveys of medical trainees and faculty, little research and none in Canada examined medical curriculum for WH. This study assessed how Canadian medical schools integrate WH in their curriculum.

**Methods:**

We used deductive and summative content analysis to describe instances and the nature of WH topics in program and course descriptions that were publicly-available on web sites of Canadian medical schools. We reported results using summary statistics and text examples. We employed a framework, tested in our prior research, that included mention of women’s health principles and practices relevant to any health concern or condition including factors (e.g. sex, gender, social determinants) that influence health, and access to or quality of care.

**Results:**

We retrieved 1459 documents from 16 medical schools (median 49.5, range 16 to 301). Few mentioned WH (125, 8.6 %), and the quantity of mentions varied by school (range 0.0–37.5 %). Pre-clerkship course documents more frequently mentioned WH (61/374, 17.3 %, chi square 43.2, *p* < 0.00001) compared with clerkship course documents (58/1067, 5.4 %). Core course documents more frequently mentioned WH (72/542, 13.3 %, chi square 29.0, *p* < 0.00001) compared with elective course documents WH (47/899, 5.2 %). Overall, documents more frequently referred to the WH domain of social determinants of health (88, 70.4 %). Few documents addressed women’s health (21, 16.8 %), sex or gender (19, 15.2 %), other considerations (15.2 %) or principles/components of women’s health (2, 1.6 %). Most documents that mentioned WH provided little detail about what those concepts referred to or how to optimize WH.

**Conclusions:**

Based on program and course descriptions, WH may not be well-integrated at Canadian medical schools, and future physicians may not be consistently exposed to the full breadth of WH. This reveals opportunities for enhancing WH in the medical curriculum. Future research is needed to engage stakeholders including women in developing, implementing and evaluating competencies and corresponding curriculum that reflect the full range of WH concepts and practices.

**Supplementary Information:**

The online version contains supplementary material available at 10.1186/s12909-021-02873-8.

## Background

Women’s health (WH) refers to a life-course approach for promoting wellness, and preventing and managing a broad array of health concerns and challenges that affect women during and beyond their reproductive years including the impact of social determinants on women’s health, morbidity and mortality, and gendered healthcare inequities [1]. Organizations such as the United Nations and World Health Organization, American WH experts, and 200 + Canadian women and WH experts have long-advocated for improving access to and quality of care for women [2–6].

Considerable population-based research shows that gendered disparities in health care quality remain a problem. Research in Canada between 2003 and 2012 revealed that women were less likely than men to receive ideal care for a range of conditions [7, 8]. More recent Canadian research also identified gendered disparities in care delivery and patient outcomes for various health conditions including cardiovascular disease [9–12], HIV management [13, 14], hip fracture [15], chronic obstructive pulmonary disease [16], mental illness [17, 18], and emergency surgery [19]. Another 1225 Canadian women said that truncated discussion of health issues was a frequent problem [20]. We recently interviewed Canadian women of varied age, ethnicity, province, urban or rural dwelling and health issue, who said that clinicians often ignored or dismissed their concerns or questions, resulting in confusion, frustration, anxiety, and dissatisfaction, and probable future avoidance of raising issues or seeking care [21, 22]. Findings are similar elsewhere in both developed and developing countries. For example, a national sample of American women were less likely than men to report good patient-provider communication and having medical needs addressed [23]. A narrative review of data sources and documents published between 1990 and 2013 pertaining to women in Iran revealed numerous disparities in women’s health compared with men [24].

Such gendered disparities may arise due to lack of competency in WH among physicians, which appears to stem from lack of exposure to WH in medical education. Canadian clinicians of varied specialty who we interviewed said they lacked training and skill in WH [25]. In a survey, 300 American primary care physicians rated many WH topics of interest for continuing education [26]. A survey of American academic general internal medicine physicians and family physicians revealed that few were confident in precepting a range of WH concerns [27]. To address such limitations, a cross-departmental commission of the American federal government assessed how to improve WH education, and recommended an audit of WH curricula across health professions [28]. In Canada, 90 % of program directors and 64 % of unit directors of family medicine residency programs surveyed in 1994 thought current teaching of WH issues was inadequate and had plans to increase it [29]. In 2008, a survey of third- and fourth-year students at 125 American medical schools revealed low ratings for curriculum coverage of WH and sex- or gender-specific topics [30]. In 2011, a survey administered to faculty at Canadian and American medical schools found that 70 % had no formal curriculum pertaining to the health effects of sex and gender [31]. In 2016, a national survey found that only 34.5 % of American medical students reported feeling prepared to manage sex and gender differences in healthcare [32].

Due to prevalent gendered inequities in quality of care [7–24] and lack in knowledge of WH among practising physicians [25–27], efforts are needed to improve women’s healthcare experiences and outcomes. Despite recognition among educational policy-makers and medical educators of the need to strengthen WH in medical education [28, 29], gaps in curriculum have persisted [30–32]. Limited research in Canada has investigated WH curriculum apart from the two noted surveys of medical educators in 1994 and 2011 [29, 31]. No research has assessed WH in the medical school curriculum more recently given national emphasis on the consideration of sex and gender in healthcare research and medical education [33], or by examining medical curriculum itself. The purpose of this study was to assess curriculum at Canadian medical schools for content related to WH. Identified gaps could inform future medical school curriculum planning.

## Methods

### Approach

We examined WH in medical curriculum documents using a content analysis approach [34]. More specifically, we employed manifest content analysis, which refers to qualitative and/or quantitative description of explicit content as reported in written communication without analyzing its underlying meaning or generating theory [35]. We employed both deductive and summative content analysis procedures to first organize and describe content in categories (deductive), and then count and compare categories included in curriculum across medical schools (summative) [34, 35]. With no reporting criteria specific to content analysis, we complied with the Standards for Reporting Qualitative Research [36]. We did not need ethics review board approval because data were publicly available.

### Eligibility

We included undergraduate medical education curriculum documents published in English or French language on the web sites of medical schools listed by the Association of Faculties of Medicine of Canada (https://afmc.ca/en/faculties). Documents included information on web pages, or files that could be viewed online or downloaded. Curriculum referred to program overviews (outline of aims, themes and competencies of the entire four-year undergraduate medical program) and course descriptions (detailed outline of weekly subjects covered, learning objectives, and projects/tests for core or elective courses) across pre-clerkship (years 1 and 2) and clerkship (years 3 and 4). For clerkship, course descriptions included in-class coursework or workshops, self-directed modules, and what would be practiced in rotations. We excluded course summaries, typically 3 to 5 sentences, opting to rely on more detailed course descriptions.

### Searching and screening

We used navigation options on the web site of each school to browse for curriculum documents. We saved all potentially-relevant documents as screen captures or downloaded files. We also contacted the dean of undergraduate medical education at each school by email to briefly introduce the purpose of our study, assure them that school identity would remain anonymized, and ask them or a designate to send us additional curriculum documents not already available on their web site. We sent an initial request between May 8 and 13, 2019, and a reminder email on both May 16 and May 21, 2019. As a pilot test, NA (woman, Research Assistant, Master of Public Health) and ARG (woman, Scientist and Professor, experienced in content analysis) together reviewed all documents acquired from one school and discussed eligibility. Thereafter, NA acquired and screened documents for all remaining schools, and consulted with ARG weekly to discuss and resolve uncertainties. Additional File 1 lists all documents acquired.

### Data extraction

For each school, we collected document details: type of document (program overview, course description), training year (1 to 4), and core or elective course. Additional File 2 includes definitions and guidance that we used to identify and extract information on WH, along with corresponding exemplar content extracted from included documents to illustrate how we operationalized components of the analytic framework. Although we were primarily interested in WH, our analytic framework encompassed a broad range of factors associated with health care disparities including gender (roles, behaviors, identities and expressions of women, men and gender diverse people) and determinants of health/access to health services (e.g. income, employment, education, ethnicity, culture, religion, etc.). With respect to the WH component of the analytic framework, our prior scoping review identified an absence of frameworks or models of women’s health [37]. Therefore, we employed a broad, inclusive approach to identify any mention of women’s health (e.g. diagnosis and treatment of diseases and conditions that affect women’s emotional and physical well-being) or considerations when delivering or supporting the care of women for any health issue [1]. This analytic framework was tested in our prior research involving content analysis of Canadian government policies and clinical guidelines for information relevant to WH [38, 39]. As a pilot test, NA and ARG together reviewed and extracted content on WH from two documents of one school. Thereafter, NA extracted data from all remaining documents, and consulted with ARG weekly to discuss and resolve uncertainties.

### Data Analysis

We used summary statistics to describe the number and type of documents; and mention of WH by school, type of document, type of course and WH domain. We used exemplar quotes to illustrate how WH was described, contrasting less- and more-detailed examples.

## Results

### Participants

Of 17 schools contacted to request documents supplemental to those on their web site, 5 (29.4 %) supplied a total of 8 documents. Of the remaining 12 (70.6 %) schools, 6 acknowledged the request but did not send documents, 2 explicitly declined, and 4 did not respond. One school did not feature publicly-available documents and did not respond to requests for documents. Overall, we included 16 of 17 Canadian medical schools.

### Search results

We retrieved 1,459 documents including 18 (1.2 %) program overviews and 1,441 (98.8 %) course descriptions, of which 542 were core and 899 were elective courses (Table 1). Across the four program years, this included 252, 122, 713 and 354 documents, respectively. By school, the median number of documents was 49.5 (range 16 to 301).

**Table 1:** Number and type of curriculum documents acquired by medical school

School	Program overview (n)	Course descriptions (n)								Subtotal n (%)
		Pre-clerkship				Clerkship				
		Year 1		Year 2		Year 3		Year 4		
		C	E	C	E	C	E	C	E	
ME001	1	6	1	10	3	11	0	0	0	32 (2.2)
ME002	1	3	0	3	0	14	0	2	1	24 (1.6)
ME003	1	7	38	8	3	8	93	5	1	164 (11.2)
ME004	2	6	4	6	4	11	4	3	9	49 (3.4)
ME005	1	4	0	1	0	10	0	0	0	16 (1.1)
ME006	1	10	1	10	0	2	6	1	51	82 (5.6)
ME007	1	36	5	0	0	16	243	0	0	301 (20.6)
ME008	1	23	0	11	1	18	1	18	5	78 (5.3)
ME009	1	17	11	6	1	14	99	1	0	150 (10.3)
ME010	1	20	0	19	0	17	92	24	85	258 (17.7)
ME011	1	10	1	9	1	5	0	2	21	50 (3.4)
ME012	1	3	0	3	0	11	0	2	6	26 (1.8)
ME013	1	20	0	0	0	10	0	2	88	121 (8.3)
ME014	2	9	0	6	0	8	2	0	0	27 (1.9)
ME015	1	10	1	9	1	11	1	1	0	35 (2.4)
ME016	1	6	0	6	1	6	0	12	14	46 (3.2)
Subtotal	18	190	62	107	15	172	541	73	281	1,459

*C* core; *E* elective

### Women’s health instances

Of 1,459 documents, 125 (8.6 %) referred to at least one analytic framework domain at least once: 19 (15.2 %) mentioned sex/gender, 21 (16.8 %) mentioned WH and 88 (70.4 %) mentioned determinants of health (Table 2). Schools varied in proportion of documents referring to WH or related concepts from 0.0 to 37.5 %. Few (5, 31.3 %) schools featured WH in program overviews. Pre-clerkship course documents more frequently mentioned WH (61/374, 17.3 %, chi square 43.2, p < 0.00001) compared with clerkship course documents (58/1067, 5.4 %). Core course documents more frequently mentioned WH (72/542, 13.3 %, chi square 29.0, *p* < 0.00001) compared with elective course documents WH (47/899, 5.2 %). Overall, documents more frequently referred to the WH domain of social determinants of health (88, 70.4 %). Few documents addressed women’s health (21, 16.8 %), sex or gender (19, 15.2 %), other considerations (15.2 %) or principles/components of women’s health (2, 1.6 %). Four schools addressed four of the five WH domains at least once.

**Table 2:** Documents referring to women’s health domains by medical school

Medical school	Documents (program overviews and course descriptions)							Women’s health domains (n of 5)
	Total (n)	Refer to women’s health n (%)	Women’s health domain n (% of 125)					
			Sex or gender	Women’s health	Determinants of health	Principles	Other considerations	
ME001	32	4 (12.5)	0	2	3	0	0	2
ME002	24	9 (37.5)	5	2	5	0	1	4
ME003	164	21 (12.8)	3	1	18	0	0	3
ME004	49	8 (16.3)	4	2	4	0	1	4
ME005	16	1 (6.25)	1	0	1	0	0	2
ME006	82	19 (23.2)	0	1	14	0	12	3
ME007	301	0 (0.0)	0	0	0	0	0	0
ME008	78	4 (5.1)	0	1	3	0	0	2
ME009	150	12 (8.0)	0	4	8	0	0	2
ME010	258	6 (2.3)	0	1	5	0	0	2
ME011	50	9 (18.0)	0	0	5	0	4	2
ME012	26	6 (23.1)	4	2	3	0	1	4
ME013	121	5 (4.1)	0	1	4	0	0	2
ME014	27	2 (7.4)	1	1	1	0	0	3
ME015	35	9 (25.7)	1	1	7	1	0	4
ME016	46	10 (21.7)	0	2	7	1	0	3
Total	1,459	125 (8.6)	19 (15.2)	21 (16.8)	88 (70.4)	2 (1.6)	19 (15.2)	---

### Women’s health details

While 8.6 % of documents referred to at least one analytic framework domain at least once, many of those documents provided little detail about what this referred to and we found few examples of such descriptions. This finding did not vary by school. Table 3 provides brief and more detailed descriptions captured from those documents. For instance, a brief example for the domain of sex and gender was: “Define sex and gender based medicine” (ME001), compared with a more detailed example of this domain: “Describe the differences between gender identity, gender expression, sexual orientation, and biological sex, and recognize that these parameters occur on a spectrum within the human population” (ME002). Similarly, a brief example of the domain of determinants of health was: “Explain how the differential distribution of health determinants influences health status” (ME002) compared with a more detailed example: “The competent graduate recognizes the diverse factors that influence the health of the individual and the community; identifies the sociocultural, familial, psychological, economic, environmental, legal, political and spiritual factors impacting health care and health care delivery; and responds to these factors by planning and advocating the appropriate course of action at both the individual and the community level” (ME005).

**Table 3:** Examples of how women’s health was described in curriculum documents

Women’s health domains	Women’s health descriptions in medical curriculum documents	
	Brief	Detailed
Sex or Gender *Biological and physiological attributes* / *Socially-constructed roles, identities and behaviours that influence health and health care*	Define sex and gender based medicine (ME001) Describe the difference between the concepts of sex and gender (ME002) Topics addressed will include: Gender and health (ME005)	Describe the differences between gender identity, gender expression, sexual orientation, and biological sex, and recognize that these parameters occur on a spectrum within the human population (ME002) Develop collaborative and respectful relationships that demonstrate gender, cultural and age related awareness and respect (ME003) This lecture will review the Global Burden of Disease and how this relates to priority setting and allocation of funds for all health-related activities and introduce the major causes of morbidity and mortality and how health risks vary by gender and income across regions (ME012)
Women’s health *Preventing, screening, and managing conditions that are unique, more common, more serious or manifest differently in women*	A broad exposure to women’s health and focus on the practice of obstetrics and gynaecology (ME001) Lecture Topic: Insomnia – different presentation in women discussed (ME004) Common Women’s Health topics to be covered by clinical exposure supplemented by independent study (ME012)	The major goals of the clerkship are to introduce students to the broad range of skills and knowledge encompassed in the specialty of obstetrics and gynecology; to demonstrate the interrelationship of specialty and primary care in the care of women across the life span; to provide students with the ability to address common inpatient and outpatient health problems of women; and to demonstrate the obstetrician-gynecologists’ interactions with other providers of medical care to achieve optimal benefit in the care of women (ME008) Clinical sciences applicable to situations in women’s health: main signs and symptoms, habits of life, paraclinical indices relevant to the differential diagnosis, risk factors and current measures of screening and preventive interventions, lifestyle and stages of change, safety of the patient and general principles of the treatments as well as the basic principles of their judicious use, their indications, contraindications and side effects related to the frequent clinical entities in the young adult (ME010)
Determinants of health *Socio-cultural factors that influence health and access to health care, including gender and other factors*	The determinants of health and the distribution of disease within and between populations will be explored (ME001) Explain how the differential distribution of health determinants influences health status (ME002)	These various disciplinary perspectives provide critical insights into human and socio-cultural dimensions of illness and healthcare experiences, which can help to foster understanding, care and compassion, self-reflection, action and advocacy (ME003) The competent graduate recognizes the diverse factors that influence the health of the individual and the community; identifies the sociocultural, familial, psychological, economic, environmental, legal, political and spiritual factors impacting health care and health care delivery; and responds to these factors by planning and advocating the appropriate course of action at both the individual and the community level (ME005) This course will include social medicine, population health, epidemiology, medical ethics and service learning and will provide students with an understanding of cultural and social roots, social inequalities, factors affecting treatment outcomes, ethical challenges, and experiential learning opportunities (ME006) Understand some of the political, social, economic and behavioral issues that influence the health of populations and influence decisions at the level of individual and societal. The student is able to recognize the cultural dimensions that influence the care of its patients and develops strategies to bypass cultural barriers that may affect the relationship. The student recognizes the influence of social determinants on the ability of his or her patients to influence their health status and proposes solutions to take into account the adverse effects of certain determinants (ME009) Know the determinants of health and their impacts, interpret data on determinants and health status, recognize its role as a physician and know the role of the partners involved in promoting the health of populations, know the main strategies for promoting health and justify the choice based on context, based on the interpretation of evidence and considering vulnerable populations, understand the ethical principles and benchmarks that guide health promotion activities (ME010) Describe the impact of the important determinants of health, the risk factors for illness, the interaction between the population and their physical, biological and social environments, and personal attributes, including: employment, income, social status, culture, social support systems, education, housing, diet and exercise, lifestyle issues, sexuality, gender, genetics (ME012) Discuss a framework for applying social determinants of health (Include topics such as absolute and relative poverty, urbanization, crowding, inadequate housing, education (especially for females), gender and other inequities, and discrimination based on race, ethnicity or other social determinants.) (ME014) Students are empowered to consider the interrelationships of culture and society on the health-care system in particular regions and demographics with professional aplomb and strong strategies for long-term care (ME015)
Delivery of care to women *Considerations (e.g. privacy, culture, trauma)*	Demonstrate a sensitive approach to all examination of intimate anatomy so as to preserve patient dignity and respect (ME004)	Demonstrate a safe and comfortable communication style and sensitive approach throughout the patient encounter, using sensitive and appropriate language, history taking skills, eye contact, gestures, body language, privacy, draping techniques and providing sensitive feedback and ongoing explanations as the exam proceeds (ME012)

## Discussion

This study aimed to assess how medical school prepares future physicians for WH by examining how WH was featured in the curriculum at 16 medical schools. Few program overviews and course documents referred to WH, although this varied from 0.0 to 37.5 % across medical schools. Significantly more pre-clerkship compared with clerkship, and core compared with elective course descriptions mentioned WH. In documents that addressed WH, the domain of social determinants of health was far more common compared with other related domains pertaining to the principles and practice of WH, sex and gender, or other considerations when caring for women. However, most documents that mentioned WH or related concepts provided little detail, and this did not vary by school. Overall, based on program overviews and course descriptions, medical students may not be consistently or thoroughly exposed to the concepts and practices of WH, or health care concerns or disparities more broadly pertaining to sex and gender.

Prior research on WH focused on women in medicine, and investigated gendered inequities in medical education and medical practice including sexual harassment, wage gaps and leadership opportunities [40]. Other prior studies analyzed undergraduate or graduate medical curriculum for WH topics; however, those studies focused on issues pertaining to reproductive health or women-specific conditions. For example, a survey of medical students at one university in Poland revealed they knew little about stress urinary incontinence [41]. A survey of family medicine, internal medicine, and obstetrics and gynecology trainees at 20 American residency programs revealed that few knew about key topics in menopause management [42]. A survey of 112 internal medicine residency program directors revealed that WH topics including sexual and reproductive health and gender-specific cancer were largely addressed in women’s health electives [43]. In Canada, content analysis of medical curriculum at one university found that obstetrics and gynecology competencies were largely covered in an obstetrics and gynecology third year clerkship [44]. This suggests that students may only be exposed to WH topics in a third year course specifically dedicated to such topics, possibly lacking exposure in their pre-clerkship years and other courses in general. Our study supports these findings by confirming that WH topics are not well-addressed in medical curriculum. Our study is unique from these studies because we employed a broader view of what constitutes WH, thereby revealing the need to enhance WH content not only in obstetrics and gynecology courses but throughout the curriculum, as well as content addressing the need to individualize care more broadly across sex and gender.

Resources are available to assist medical educators who oversee or implement medical curriculum in developing WH content. For example, the American Association of Professors of Gynecology and Obstetrics participated in translating competencies that undifferentiated medical students should be able to demonstrate before graduation into educational objectives, and the level of professional competence medical students should reach for each objective [45]. Other universities have developed WH curriculum that could be broadly emulated. The University of Florida developed a two-week elective undergraduate course on WH topics such as disease prevention, screening, breast health, osteoporosis, and cardiovascular health including case-based lectures, reading assignments, and clinic sessions at multiple sites, and showed that it significantly improved student knowledge [46]. West Virginia University developed a one-month internal medicine residency rotation in WH topics such as uro-gynecology and breast cancer including work in a variety of clinical settings and web-based tutorials, and showed that it significantly improved knowledge, and the majority of students said the rotation increased their awareness of and skills in caring for women [47]. These two initiatives relied on published research and local expertise to develop courses. At the University of Texas, a multidisciplinary task force representing five primary care medical specialties developed a WH curriculum for medical students and residents using a three-round Delphi method to prioritize topics identified by literature review, and generate corresponding activities and skills [48]. Topics mainly reflected reproductive health and women’s cancers but also included health promotion, cardiovascular care, musculoskeletal disorders, mental health and the caregiver role.

While such resources are helpful to future curriculum planning and improvement, curriculum largely focused on reproductive health. Similarly, a scoping review of WH curriculum in North American internal medicine residency programs that included 16 studies revealed that the most common WH topics were intimate partner violence and menopause [49]. In contrast, it is widely agreed that WH is much broader than domestic violence or reproductive health [1]. Medical education must expand to include a range of healthcare conditions, and beyond the lifespan context to consider not only healthcare conditions, but also issues and challenges that affect women’s health and health care. Considerable research has established wide-spread gendered inequities in access to and quality of care [7–19, 23, 24], further exacerbated by social determinants of health such as race, ethnicity, culture, education and employment, and other research shows that women’s healthcare concerns and questions are often dismissed [20–22]. One way to address these problems is to ensure that future generations of physicians are aware of these issues and understand what can be done at the individual, organizational and system levels to prevent and mitigate such inequities. At the same time, further research is needed to establish competencies that reflect the full range of WH topics; develop, implement and evaluate that curriculum; and explore awareness, attitudes and knowledge among educators that establish and implement medical curriculum. Another parallel and fundamental approach to strengthening WH in the medical curriculum is to do so in collaboration with women, as patient engagement has become a standard practice in research, and in health system planning and improvement [50, 51].

Strengths of this research included use of rigorous methods and adherence to reporting standards [34–36]. We also analyzed curriculum for a large number of medical schools. Still, we must acknowledge some limitations. We analyzed publicly-available course descriptions, which may not reflect potentially more substantive content about WH taught in those courses. Although we analyzed curriculum for 16 of 17 Canadian medical schools, the findings may not necessarily reflect how WH is integrated in curriculum at medical schools in other countries. To address these limitations, future research is warranted to assess WH in course content, possibly through field observation, or qualitative interviews with students or faculty, and to assess WH content in the curriculum of medical schools in jurisdictions other than Canada. While our primary focus was WH, our analytic framework encompassed sex and gender, and revealed little such content, suggesting the need for future research to adopt a broad lens and examine how medical curriculum could address the need for individualized care. Based on the results of this research, ongoing investigation should explore barriers of integrating WH into an already full medical curriculum. This could be explored by interviewing medical students, recently-licensed physicians, academics who specialize in medical education, and deans of undergraduate and graduate medicine. We aimed to describe mention of WH and related concepts in medical curriculum, but we did not examine the language used to describe those concepts. Gender neutral or inclusive language is now considered desirable to equally value and respect gender diversity. Given variations in knowledge and attitudes about gender-inclusive/-sensitive language [52, 53], ongoing research is needed on how to provide training in both medical and continuing education.

## Conclusions

Practising physicians self-report lacking knowledge and skill in WH, which may, in part, contribute to gendered inequities in access to and quality of care, and sub-optimal healthcare experiences for many women in both developed and developing countries. One way to address this is by strengthening exposure to WH in medical school. Our study of program and course descriptions at 16 Canadian medical schools showed that, based on the narrow scope of curricula provided, WH may not be well-integrated. This reveals opportunities for enhancing student exposure to WH in the medical curriculum. Future research is needed to confirm these findings, which are based on program overviews and course descriptions, and engage stakeholders including women in developing, implementing and evaluating competencies and corresponding curriculum that reflect the full range of WH concepts and practices.

## Supplementary Information

**Additional file 1:** Curriculum documents. Tables listing all program overview and course documents retrieved from 16 medical school web sites 

**Additional file 2:** Guidance for extracting WH data. Definitions and guidance for WH data extraction. Table listing WH domains, descriptions, extraction criteria, and brief and more detailed examples extracted from documents to illustrate domains 

## Data Availability

All data generated or analysed during this study are included in this published article and its supplementary information files.

## Abbreviations

WH: Women’s health
