# Supplementary material for: Medical student exposure to women’s health concepts and practices: a content analysis of curriculum at Canadian medical schools
Source: BMC Med Educ. 2021 Aug 18;21:435. doi: 10.1186/s12909-021-02873-8 (PMC8371837; doi:10.1186/s12909-021-02873-8)
Supplement: Supplementary file 1 — Additional file 1: Curriculum documents. Tables listing all program overview and course documents retrieved from 16 medical school web sites [file 12909_2021_2873_MOESM1_ESM.docx]

**Medical student exposure to women’s health concepts and practices: A content analysis of curriculum at Canadian medical schools**

Natalie N. Anderson, MPH, Anna R. Gagliardi, PhD, Toronto General Hospital Research Institute, University Health Network, Toronto, Canada

**Additional File 1. Curriculum documents**

NOTE: Courses offered in either Year 1 or Year 2, or in Year 3 or Year 4 appear in Year 1 and Year 3, respectively

| School | Program overview | Pre-clerkship | | Clerkship | |
| --- | --- | --- | --- | --- | --- |
|  |  | Year 1 | Year 2 | Year 3 | Year 4 |
| ME001 | 1 document | **Core**  Medical Skills (Medicine 320)  Family Medicine Clinical Experience (Medicine 330)  Population Health (Medicine 340)  Introduction to Medicine, Blood and Gastrointestinal Course (Medicine 350)  Integrated Musculoskeletal and Dermatology Course (Medicine 360)  Integrated Cardiovascular and Respiratory Course (Medicine 370)  **Elective**  Applied Evidence-Based Medicine I (Medicine 345) | **Core**  Second Year Elective (Medicine 402)  Integrated Renal-Electrolyte and Endocrine-Metabolic Course (Medicine 410)  Medical Skills (Medicine 420)  Family Medicine Clinical Experience (Medicine 430)  Applied Evidence-Based Medicine (Medicine 440)  Integrated Neurosciences, Special Senses and Aging Course (Medicine 450)  Children and Women’s Health (Medicine 460)  Psychiatry (Medicine 470)  Integrative Course I (Medicine 480)  Introduction to Clinical Practice I (Medicine 490)  **Elective**  Applied Evidence-Based Medicine II (Medicine 445)  Integrative Course II (Medicine 485)  Introduction to Clinical Practice II (Medicine 495) | **Core**  Family Medicine (Medicine 502)  Internal Medicine (Medicine 504)  Surgery (Medicine 506)  Paediatrics (Medicine 508)  Psychiatry (Medicine 510)  Obstetrics and Gynaecology (Medicine 512) [+1 additional file provided by school]  Clerkship Electives (Medicine 514)  Extended Clerkship Electives (Medicine 515)  Anaesthesia (Medicine 516)  Comprehensive Clinical Skills Curriculum for Clerkship (Medicine 520)  Emergency Medicine (Medicine 522)  **Elective** – no documents | (only a 3 year program) |
| ME002 | 1 document | **Core**  Introduction to Medicine (MED100H) [+1 additional file provided by school (A)]  Concepts, Patients & Communities 1 (MED120H) [+1 additional file provided by school (A)]  Concepts, Patients & Communities 2 (MED130H) [+1 additional file provided by school (A)]  **Elective**  None offered | **Core**  Concepts, Patients & Communities 3 (MED200H) [+1 additional file provided by school (A)]  Life Cycle (MED210H) [+1 additional file provided by school (A)]  Complexity and Chronicity (MED220H) [+1 additional file provided by school (A)]  **Elective**  None offered | **Core**  Transition to Clerkship (TTC310Y)  Anesthesia (AND310Y)  Dermatology (DER310Y)  Emergency Medicine (EMR310Y)  Family & Community Medicine (FCM310Y)  Internal Medicine (MED310Y)  Obstetrics & Gynaecology (OBS310Y) [+1 additional file provided by school (B)]  Ophthalmology (OPT310Y)  Otolaryngology – Head & Neck Surgery (OTL310Y)  Paediatrics (PAE310Y)  Psychiatry (PSS310Y)  Surgery (SRG310Y)  Portfolio – Year 3 (PFL310Y)  Elective  Integrated Objective Structured Clinical Examination (OSC310Y & OSC410Y)  **Elective**  None offered | **Core**  Portfolio – Year 4 (PFL410Y)  Transition to Residency (TTR410Y)  **Elective**  Electives (ELV410Y) |
| ME003 | 1 document | **Core**  Foundations of Medicine and Dentistry (MED 511)  Endocrine System (MED 513)  Cardiovascular System (MED 515)  Pulmonary System (MED 516)  Renal System (DMED 517)  Physicianship I (MED 516)  Interprofessional Health Team Development (INT410)  **Elective**  First Year Elective (MED 517)  Optional Summer Elective (MED518)  Art in Medicine (AIM) Project: Pre-Clerkship Elective  **Elective (Year 1 or 2)**  Anesthesia Medicine  Cardiovascular Anesthesia  Directed Studies in Medical/Health Humanities: Pre-Clerkship Elective  Communicating Care: A Theatre-Based Approach  Introduction to Mindfulness - Section 1  Introduction to Mindfulness (Winter/Spring, 2019) - Section 2  Mindfulness: Advances Practice  Shadowing Artists on the Wards: Promoting Patient-Centredness through an Arts-Based Medical Elective  Spiritual Screening and Assessment: A Communication Skills Workshop  The Art of Observation: Learning to See  The Healer's Art Elective:  Remembering the Heart of Medicine  Organ/Tissue Transplantation  Community Service Learning  Health Advocacy  Indigenous Health  Inner City Health  International Health  Community Engagement  Sexual and Reproductive Health  Emergency Department Experience  Medical Ethics: An Introduction  Family Medicine  Fundamentals of Patient Safety & Quality Improvement  International Comparisons in Health Systems and Health Services  Measuring the Quality and Capacity of Medical Services  Genetics  Laboratory Medicine  Technology and the Future of Medicine (LABMP590)  Microbiology  HIV  Infectious Disease: Consultation Experience  Developing Skills for Developmental Disabilities  Pediatric Cardiology  General Surgery  Specialty Surgery | **Core**  Gastroenterology & Nutrition (MED 521/DDS 506)  Reproductive Medicine & Urology (MED 522)  Musculoskeletal System (MED 523/DDSMedicine 523)  Psychiatry (MED 529)  Neurosciences and Organs of Special Senses (MED 524/DDS 507)  Oncology (MED 525/DDS 508)  Physicianship II (MED 526)  Pre-Clinical Exam (MED 5210)  **Elective**  Second Year Elective (F MED 527)  Optional Summer Elective (MED 528)  Spirituality and Health: Spiritual Care Shadowing Elective | **Core**  Physicianship III /Transitions (MED 531)  Integrated Community Clerkship (MED 543)  Medicine (MED 546)  Obstetrics & Gynecology (OB GY 546)  Pediatrics (PAED 546)  Psychiatry (PSYCI 546)  Family Medicine (F MED 546)  General Surgery (SURG 546)  **Elective**  Clinical Electives (MED 547)  **Elective (Year 3 or 4)**  Genetics  Anatomy  Clinical Anesthesiology  Art in Medicine (AIM) Project: Clerkship Elective  Directed Studies in Medical/Health Humanities: Clerkship Elective  Medical/Health Humanities: “Mix and Match” Clerkship Elective  Emergency Medicine  Community Emergency Medicine  An Experience in Bioethics  Evidence Based Medicine – Research  Inner City & Addictions Medicine  International Traditional Chinese Medicine  Northern Medicine – Inuvik  Primary Care Hospital Team – PCHT  Primary Care Obstetrics  Rural Family Practice  Sports and Exercise Medicine  Urban Family Medicine  Geriatric Medicine and Care of the Elderly  General Pathology–RAH  Anatomical/General Pathology  Hematological Pathology  Medical Microbiology  Neuropathology  Research in Medical Microbiology  Sudden Death Investigation  Adult Neurology  Clinical Cardiology – UAH  Clinical Cardiology - Cardiac Intensive Care Unit (CICU) – RAH  Clinical Endocrinology and Diabetes  Adult Clinical Hematology  Clinical Pulmonary Disease  Dermatology  Gastroenterology  General Internal Medicine ED Consultations Service  General Internal Medicine Ward Consultation Service  Infectious Disease  Nephrology  Occupational Medicine  Rheumatology  Wilderness Medicine/Environmental Medicine  Clinical Obstetrics and Gynecology  Clinical Obstetrics and Gynecology with a focus in Maternal Fetal Medicine  Subspecialty Gynecology  Introduction to Clinical Oncology Practice  Palliative Care  Radiation Oncology  Ophthalmology  Developmental Pediatrics  Neonatology (NICU)  Pediatric Allergy & Clinical Immunology  Pediatric Cardiology  Pediatric Emergency  Pediatric Endocrinology  Pediatric Gastroenterology  Pediatric Hematology  Pediatric Infectious Diseases  Pediatric Integrative Medicine (PIM) clinical elective  Pediatric Intensive Care (PICU)  Pediatric Nephrology  Pediatric Neurology  Pediatric Oncology  Pediatric Pulmonology  Pediatric Rehabilitation Medicine  Physiatry/rehabilitation  Adolescent Forensic Inpatient Unit  Child and Adolescent Psychiatry in Acute, Chronic and Office Space Settings  Community Child and Adolescent Psychiatry  Community Psychiatry  Emergency Psychiatry  Evening Treatment Program for Patients with Personality Disorder and Comorbid Axis I Disorders  Geriatric Psychiatry  Introduction to Forensic Psychiatry  Medical Surgical Psychiatry (Consultation Liaison)  Psychiatric Out Patient Program  Day Treatment Program  Transgender Mental Health  Public Health  Diagnostic Radiology at GNH  Diagnostic Radiology at MIS  Diagnostic Radiology at the RAH  Diagnostic Radiology at the UAH  Nuclear Medicine UAH or Nuclear Medicine RAH  Cardiac Surgery  General Surgery  Neurosurgery  Orthopedic Surgery  Otolaryngology - Head & Neck Surgery  Plastic & Reconstructive Surgery  Surgical Education  Urology  Vascular Surgery | **Core**  Physicianship IV /Transitions (MED 541)  Geriatrics (MED 555)  Internal Medicine (MED 556)  Emergency Medicine (MED 558)  Specialty Surgery (SURG 556)  **Elective**  Clinical Electives (MED 557) |
| ME004 | 1 document [+1 additional file provided by school (A)] | **Core**  Community Week (Med 1107)  Introduction Unit to the Profession (MED 1200)  Foundations Unit (MED 1201)  Unit 1 (MED 1202)  Physician Skills Development (Part 1) (MED 1302) [+1 additional file provided by school (A)]  E-portfolio on Core Competencies (Part 1) (MED 4101)  **Elective**  Clinical Placement (ELE 1900)  Clinical Placement (ELE 19001)  Clinical Placement (ELE 19002)  Clinical Placement (ELE 19003) | **Core**  Mandatory Clinical Week (MED 2109)  Unit II (MED 2201)  Unit III (MED 2202)  Integration Unit (MED 2203)  Physician Skills Development (Part 2) (MED 2302) [+1 additional file provided by school (A)]  Eportfolio on Core Competencies (Part 2) (MED 4102)  **Elective**  Clinical Placement (ELE 2900)  Clinical Placement (ELE 29001)  Clinical Placement (ELE 29002)  Clinical Placement (ELE 29003) | **Core**  Link Period (CLI 3101)  Surgery (CLI 3102)  Internal Medicine (CLI 3103)  Mandatory Selectives (CLI 3104)  Obstetrics and Gynecology (CLI 3102) [+1 additional file provided by school (A)]  Pediatrics (CLI 3107)  Psychiatry (CLI 3108) [+1 additional file provided by school (A)]  Family Medicine (CLI 3109) [+1 additional file provided by school (A)]  Acute Care Medicine (CLI 3110)  Objective structured Clinical Examination (CLI 3111)  Eportfolio on Core Competencies (Part 3) (MED 4103)  **Elective**  Year of Enrichment (ELE 3951)  Year of Enrichment (ELE 39511)  Year of Enrichment (ELE 39512)  Year of Enrichment (ELE 3953) | **Core**  Back to Basics (CLI 4105)  Selective in Ambulatory Medicine (CLI 4106)  Eportfolio on Core Competencies (Part 4) (MED 4104)  **Elective**  Electives (CLI 4104)  Year of Enrichment (ELE 4931)  Year of Enrichment (ELE 49311)  Year of Enrichment (ELE 49312)  Year of Enrichment (ELE 49313)  Clinical Placement for Visiting Medical Students (Canadian Schools) / Clinical Placement for Visiting Medical Students (Canadian Schools) (ELE 4951)  ELE 4952 Clinical Placement for Canadian School Visitors / Optional Rotation for Visiting Trainees from Canadian Schools (ELE 4952)  Elective for Canadians Studying Abroad Internship (ELE 4961)  Clinical Placement for Non-Canadian School Visitors / Optional Rotation for Visiting Trainees from Non-Canadian Schools (ELE 4962) |
| ME005 | 1 document | **Core**  Medical Foundation 1  Medical Foundation 2  Medical Foundation 3  Medical Foundation 4  **Elective –** no documents | **Core**  Medical Foundation 5 Elective - no documents | **Core**  [Anesthesia](javascript:__doPostBack('ctl00$cphbd$C003$ctl00$ctl00$ctl00$listsControl$ctrl0$listItemsControl$ctrl0$listItemToggleLnk','')) [Emergency Medicine](javascript:__doPostBack('ctl00$cphbd$C003$ctl00$ctl00$ctl00$listsControl$ctrl0$listItemsControl$ctrl1$listItemToggleLnk','')) Concept Integration and Review (CIR) [Family Medicine](javascript:__doPostBack('ctl00$cphbd$C003$ctl00$ctl00$ctl00$listsControl$ctrl0$listItemsControl$ctrl2$listItemToggleLnk','')) [Internal Medicine](javascript:__doPostBack('ctl00$cphbd$C003$ctl00$ctl00$ctl00$listsControl$ctrl0$listItemsControl$ctrl3$listItemToggleLnk','')) [Obstetrics and Gynecology](javascript:__doPostBack('ctl00$cphbd$C003$ctl00$ctl00$ctl00$listsControl$ctrl0$listItemsControl$ctrl4$listItemToggleLnk',''))[Orthopedic Surgery](javascript:__doPostBack('ctl00$cphbd$C003$ctl00$ctl00$ctl00$listsControl$ctrl0$listItemsControl$ctrl5$listItemToggleLnk',''))[Pediatrics](javascript:__doPostBack('ctl00$cphbd$C003$ctl00$ctl00$ctl00$listsControl$ctrl0$listItemsControl$ctrl6$listItemToggleLnk',''))[Psychiatry](javascript:__doPostBack('ctl00$cphbd$C003$ctl00$ctl00$ctl00$listsControl$ctrl0$listItemsControl$ctrl7$listItemToggleLnk','')) [Surgery](javascript:__doPostBack('ctl00$cphbd$C003$ctl00$ctl00$ctl00$listsControl$ctrl0$listItemsControl$ctrl8$listItemToggleLnk',''))  **Elective -** no documents | (only a 3 year program) |
| ME006 | 1 document | **Core**  Introduction to Medicine (MED5115)  Blood (MED5121)  Infection & Immunity (MED5116)  Skin (MED5117)  Heart & Circulation (MED120)  Respiration & Airways (MED5119)  Genitourinary System (MED5104)  Social Medicine (MED5151)  Patient Centred Clinical Methods (Year 1) (MED5139)  **Core (Year 1 or 2)**  Professional Portfolio (MED5140)  **Elective**  Pre-Clinical International Health Equity Learning | **Core**  Digestive System & Nutrition (MED5203)  Endocrine and Metabolism (MED5202)  Reproduction (MED5205)  Key Topics in Family Medicine (MED5210S)  Musculoskeletal System (MED5218)  Emergency Care (MED5208)  Neurosciences, Eye & Ear (MED5206)  Psychiatry & Behavioural Sciences (MED5207)  Professional Identity (MED5250)  Patient centred Clinical Methods (MED5246)  **Elective –** none offered | **Core (Year 3 or 4)**  Clerkship (MED5475)  Clinical Science Electives (MED5401)  **Elective (Year 3 or 4)**  Anesthesia & Perioperative Medicine  Clinical Neurological Sciences – Division of Neurology  Clinical Neurological Sciences – Division of Neurosurgery  Diagnostic Radiology  Nuclear Medicine  Public Health | **Core**  Integration and Transition (Medicine 5402).  **Elective**  Emergency Medicine  Infectious Diseases  Family Medicine  Respirology  Allergy/Immunology  Cardiology  Endocrinology  Gastroenterology  General Medicine Team  Radiation Oncology  Medical Oncology  General Internal Medicine Clinical Teaching Unit  Geriatric Medicine  Hematology  Nephrology  Rheumatology  Perioperative & Ambulatory General Internal Medicine  Obstetrics & Gynaecology  General Obstetrics and Gynaecology  Gynaecology and Reproductive Medicine  Maternal/Fetal Medicine  Urogynaecology  Gynaecological Oncology  Occupational Medicine and Family Medicine Elective in Windsor  Clinical International Health Equity Learning  Ophthalmology  Otolaryngology – Head and Neck Surgery  Pediatrics & Medical Genetics – Cardiology  Pediatrics & Medical Genetics – Endocrinology  Pediatrics & Medical Genetics - Gastroenterology  Pediatrics & Medical Genetics - Hematology/Oncology,  Pediatrics & Medical Genetics – Neonatology  Pediatrics & Medical Genetics – Nephrology  Pediatrics & Medical Genetics – Neurology  Pathology – Anatomical Pathology  Pathology – Neuropathology  Physical Medicine & Rehab  Psychiatry  Surgery  Cardiac Surgery  Orthopedic Surgery  Pediatric Surgery  Plastic & Reconstructive Surgery  Thoracic Surgery  Urology Surgery  Vascular Surgery  General Surgery  Hospitalist/Palliative  Medicine – Critical Care ICU  Paediatrics – Community  Paediatrics - CTU |
| ME007 | 1 document | **Core (Year 1 or 2)**  Basic Sciences I (MED-1200)  Basic Sciences II (MED-1201)  Respiratory system: foundations and clinical problems (MED-1202)  Cardiovascular system: foundations and clinical problems (MED-1203)  Doctor, medicine and society I (MED-1204)  Clinical approach I (MED-1205)  Clinical approach 2 (part 1) (MED-1199)  Basic Sciences III (MED-1206)  Digestive system: foundations and clinical problems (MED-1207)  Urinary system and inner environment (MED-1208)  Doctor, medicine and society II (MED-1210)  Clinical approach II (part 2) (MED-1211)  Integration I (MED-1233)  Endocrine system: foundations and clinical problems (MED-1234)  Nervous system: foundations and clinical problems (MED-1216)  Integration II (MED-1220)  Epidemiology and critical reading I (MED-2231)  Sense Organs: Foundations and Clinical Problems (MED-1209)  Locomotor system: foundations and clinical problems (MED-1221)  Reproductive system: foundations and clinical problems (MED-1223)  Epidemiology and critical reading II (MED-2232)  Skin coating: foundations and clinical problems (MED-1225)  Seniors and end-of-life care (MED-1228)  Normal child and pediatric problems (MED-1230)  Psychism: foundations and clinical problems (MED-1217)  Doctor, medicine and society III  (MED-1219)  Hematopoietic system: foundations and clinical problems (MED-1222)  Doctor, medicine and society IV (MED-1229)  Integration III (MED-2200)  Person-Centered Interprofessional Collaboration 1 (FIS-4101)  Person-Centered Interprofessional Collaboration 2 (FIS-4102)  Person-Centered Interprofessional Collaboration 3 (FIS-4103)  Introduction to day school (MED-2502)  Skills development monitoring I (MED-1750)  Skills development monitoring II (MED-2750)  Skills development monitoring III (MED-2760)  **Elective (Year 1 or 2)**  Clinical approach III (MED-1290)  Clinical approach III part 1 (MED-1291)  Clinical approach III part 2 (MED-1292)  Clinical approach IV (MED-2293)  Clinical approach V (MED-2294) | **Core**  **Elective** | **Core (Year 3 or 4)**  Family medicine internship in the region (MED-3500)  Medical internship (MED-3501)  Surgery internship (MED-3502)  Internship in psychiatry (MED-3503)  Internship in elderly and end-of-life care (MED-3504)  Pediatrics internship (MED-3505)  Internship in gynecology and obstetrics (MED-3661)  Emergency internship (MED-3533)  Clinical skills (MED-3534)  Internship in diagnostic radiology (MED-3535)  Public health internship (MED-3537)  Introductory course in anesthesiology (MED-3538)  Synthesis (MED-3205)  Synthesis, integration and preparation for exams (MED-3652)  Monitoring of skills development IV (MED-3751)  Skills development monitoring V (MED-3761)  **Elective (Year 3 or 4)**  Cardiac surgery internship (MED-3511)  Internship in cardiac surgery outside the Université Laval network (MED-3512)  Internship in cardiac surgery - optional 3 (MED-3762)  Internship in general surgery outside the Laval University network (MED-3513)  Internship in pediatric surgery (MED-3514)  Thoracic surgery internship (MED-3517)  Internship in vascular surgery (MED-3518)  Surgical trauma internship (MED-3610)  Surgical intensive care internship (MED-3611)  Internship in thoracic surgery outside the Laval University network (MED-3644)  Internship in vascular surgery outside network Laval University (MED-3645)  Internship in surgical trauma outside the Université Laval network (MED-3646)  Internship in surgical intensive care outside the Laval University network (MED-3647)  General surgery internship - optional 2 (MED-3662)  Internship in pediatric surgery - optional 2 (MED-3764)  Thoracic surgery internship - optional 3 (MED-3766)  Internship in vascular surgery - optional 3 (MED-3767)  Surgical intensive care internship - optional 3 (MED-3775)  Surgical trauma internship - optional 3 (MED-3776)  Internship in pediatric surgery - optional 3 (MED-3782)  Internship in plastic surgery (MED-3515)  Internship in plastic surgery outside the Laval University network (MED-3516)  Internship in plastic surgery - optional 3 (MED-3765)  Internship in neurosurgery (MED-3519)  Internship in neurosurgery outside network Université Laval (MED-3520)  Internship in neurosurgery - optional 3 (MED-3772)  Internship in obstetrics and gynecology outside the Laval University network (MED-3521)  Internship in gyneco-oncology (MED-3613)  Risk pregnancy clinic internship (MED-3614)  Internship in obstetrics and gynecology - optional 2 (MED-3663)  At-risk pregnancy clinic - optional 2 (MED-3768)  At-risk pregnancy clinic - optional 3 (MED-3769)  Internship in gyneco-oncology - optional 2 (MED-3770)  Internship in gyneco-oncology - optional 3 (MED-3771)  Fertility internship (MED-3792)  Ophthalmology internship (MED-3522)  Internship in ophthalmology outside the Laval University network (MED-3523)  Ophthalmology internship - optional 3 (MED-3773)  Otolaryngology internship (MED-3524)  Internship in otolaryngology outside the Laval University network (MED-3525)  Otolaryngology internship - optional 3 (MED-3774)  Internship in orthopedic surgery (MED-3526)  Internship in orthopedic surgery outside network Laval University (MED-3527)  Orthopedic internship - optional 3 (MED-3763)  Urology internship (MED-3528)  Internship in urology outside network Université Laval (MED-3529)  Pediatric urology internship (MED-3615)  Internship in gynecological urology (MED-3616)  Urology internship - optional 3 (MED-3777)  Internship in gynecological urology - optional 2 (MED-3778)  Internship in gynecological urology - optional 3 (MED-3779)  Pediatric urology internship - optional 2 (MED-3780)  Pediatric urology internship - optional 3 (MED-3781)  IFMSA internship in surgery (MED-3639)  Internship in medical biochemistry (MED-3552)  Internship in medical biochemistry outside network Laval University (MED-3553)  Biochemistry internship - optional 3 (MED-3677)  Internship in dermatology (MED-3555)  Internship in dermatology outside Laval University network (MED-3556)  Surgical dermatology internship (MED-3612)  Oncology dermatology internship (MED-3617)  Internship in dermatology - optional 3 (MED-3681)  Surgical dermatology internship - optional 2 (MED-3682)  Surgical dermatology internship - optional 3 (MED-3683)  Internship in dermatology oncology - optional 2 (MED-3684)  Internship in dermatology oncology - optional 3 (MED-3685)  Cardiology internship (MED-3554)  Endocrinology internship (MED-3557)  Internship in gastroenterology (MED-3558)  Hematology internship (MED-3561)  Immunoallergology internship (MED-3562)  Internship in internal medicine outside Laval University network (MED-3566)  Nephrology internship (MED-3572)  Internship in medical oncology (MED-3575)  Pneumology internship (MED-3584)  Rheumatology internship (MED-3591)  Intensive care medical internship (MED-3597)  Geriatric internship (MED-3598)  Lipid Disease Clinic Internship (MED-3618)  Internship in obstetric medicine (MED-3619)  Internship in endocrinology outside network Université Laval (MED-3648)  Internship in gastroenterology outside the Laval University network (MED-3649)  Hematology-oncology internship (MED-3653)  Internship in nephrology outside network Université Laval (MED-3654)  Internship in pneumology outside the network Laval University (MED-3655)  Rheumatology internship outside Laval University network (MED-3656)  Intensive care internship outside the Laval University network (MED-3657)  Cardiology internship - optional 2 (MED-3664)  Geriatric internship - optional 2 (MED-3665)  Immunoallergology internship - optional 2 (MED-3666)  Internship in internal medicine - optional 2 (MED-3667)  Cardiology internship - optional 3 (MED-3678)  Endocrinology internship - optional 3 (MED-3686)  Internship in gastroenterology - optional 3 (MED-3687)  Hematology internship - optional 2 (MED-3688)  Hematology internship - optional 3 (MED-3689)  Hematology / Oncology internship - optional 2 (MED-3690)  Hematology / Oncology internship - optional 3 (MED-3691)  Immunoallergology internship - optional 3 (MED-3692)  Nephrology internship - optional 3 (MED-3696)  Internship in medical oncology - optional 2 (MED-3698)  Internship in medical oncology - optional 3 (MED-3699)  Pneumology internship - optional 3 (MED-3700)  Rheumatology internship - optional 3 (MED-3701)  Intensive care medical internship - optional 3 (MED-3702)  Internship in lipid diseases clinic - optional 2 (MED-3679)  Internship in lipid diseases clinic - optional 3 (MED-3680)  Internship in obstetric medicine - optional 2 (MED-3693)  Internship in obstetric medicine - optional 3 (MED-3694)  Internship in infectious diseases (MED-3570)  Internship in off-grid infectiology (MED-3571)  Internship in infectious diseases - optional 3 (MED-3695)  Neurology internship (MED-3573)  Internship in off-grid neurology Université Laval (MED-3574)  Neurology internship - optional 3 (MED-3697)  IFMSA internship in medicine (MED-3640)  Adolescent Medicine Internship (MED-3563)  Internship in pediatrics outside the Laval University network (MED-3578)  Internship in subspecialized pediatrics (MED-3579)  Pediatric cardiology internship (MED-3620)  Internship in pediatric endocrinology (MED-3621)  Internship in pediatric gastroenterology (MED-3622)  Pediatric hematology internship (MED-3623)  Internship in pediatric infectiology (MED-3624)  Neonatology internship (MED-3625)  Neuropediatrics internship (MED-3626)  Pediatric oncology internship (MED-3627)  Pediatric pulmonology internship (MED-3629)  Internship in pediatric dermatology (MED-3658)  Pediatric cardiology internship - optional 2 (MED-3726)  Pediatric cardiology internship - optional 3 (MED-3727)  Internship in pediatric dermatology - optional 2 (MED-3728)  Internship in pediatric dermatology - optional 3 (MED-3729)  Internship in pediatric endocrinology - optional 2 (MED-3730)  Pediatric endocrinology internship - optional 3 (MED-3731)  Internship in pediatric gastroenterology - optional 2 (MED-3732)  Internship in pediatric gastroenterology - optional 3 (MED-3733)  Pediatric hemato-oncology internship - optional 2 (MED-3734)  Pediatric hemato-oncology internship - optional 3 (MED-3735)  Pediatric infectious disease internship - optional 2 (MED-3736)  Internship in pediatric infectiology - optional 3 (MED-3737)  Adolescent medicine internship - optional 2 (MED-3738)  Adolescent medicine internship - optional 3 (MED-3739)  Neonatology internship - optional 2 (MED-3740)  Neonatology internship - optional 3 (MED-3741)  Neuropediatrics internship - optional 2 (MED-3742)  Neuropediatrics internship - optional 3 (MED-3743)  Internship in pediatric oncology - optional 2 (MED-3744)  Pediatric oncology internship - optional 3 (MED-3745)  Pediatrics internship - optional 2 (MED-3746)  Internship in subspecialty pediatrics - optional 2 (MED-3747)  Pediatric pulmonology internship - optional 2 (MED-3748)  Pediatric pulmonology internship - optional 3 (MED-3749)  IFMSA internship in pediatrics (MED-3641)  Intensive care course in pediatrics (MED-3794)  Internship in family medicine (environment different from OB internship) (MED-3565)  Palliative care internship (MED-3594)  Optional emergency internship (MED-3595)  Trauma internship (MED-3599)  Perinatal internship - optional 3 (MED-3603)  Palliative care internship - optional 3 (MED-3607)  Internship in medical trauma - optional 3 (MED-3609)  Breast disease clinic internship (MED-3631)  Perinatal internship (MED-3632)  Internship in family medicine - optional 2 (MED-3668)  Perinatal internship - optional 2 (MED-3671)  Palliative care internship - optional 2 (MED-3673)  Medical trauma internship - optional 2 (MED-3675)  Emergency internship - optional 2 (MED-3676)  Breast disease clinic internship - optional 2 (MED-3789)  Breast disease clinic internship - optional 3 (MED-3790)  Internship in occupational medicine (MED-3564)  Internship in occupational medicine - optional 3 (MED-3601)  Internship in occupational medicine - optional 2 (MED-3670)  Physiatry internship (MED-3582)  Internship in physiatry outside the Laval University network (MED-3583)  Internship in sports medicine (MED-3569)    Rehabilitation internship (MED-3589)  Internship in sports medicine - optional 3 (MED-3602)  Rehabilitation internship - optional 3 (MED-3605)  Internship in sports medicine - optional 2 (MED-3669)  Rehabilitation internship - optional 2 (MED-3672)  Community or public health internship (MED-3592)  Internship in community or public health outside Laval University network (MED-3593)  Community / public health internship - optional 3 (MED-3606)  Internship in toxicology - drug addiction - optional 3 (MED-3608)  Internship in toxicology-drug addiction (MED-3630)  Internship in toxicology - drug addiction - optional 2 (MED-3674)  IFMSA internship in family medicine (MED-3642)  Internship in anesthesiology (MED-3550)  Internship in anesthesiology outside network Université Laval (MED-3551)  Pain clinic internship (MED-3793)  Internship in medical genetics (MED-3559)  Internship in medical genetics outside the Laval University network (MED-3560)  Internship in medical genetics - optional 3 (MED-3703)  Nuclear medicine internship (MED-3567)  Internship in nuclear medicine outside the Laval University network (MED-3568)  Internship in nuclear medicine - optional 3 (MED-3706)  Internship in laboratory microbiology (MED-3638)  Internship in laboratory microbiology - optional 2 (MED-3707)  Internship in laboratory microbiology - optional 3 (MED-3708)  Pharmacology internship (MED-3712)  Pharmacology internship - optional 2 (MED-3713)  Pharmacology internship - optional 3 (MED-3714)  Pathology internship (MED-3576)  Internship in pathology outside the Laval University network (MED-3577)  Neuropathology internship (MED-3633)  Neuropathology internship - optional 2 (MED-3709)  Neuropathology internship - optional 3 (MED-3710)  Pathology internship - optional 3 (MED-3711)  Internship in psychiatry outside the Université Laval network (MED-3580)  Internship in subspecialized psychiatry (MED-3581)  Internship in child psychiatry (MED-3628)  Internship in gerontopsychiatry (MED-3634)  Internship in personality disorders (MED-3635)  Internship in forensic psychiatry (MED-3636)  Internship in forensic psychiatry (MED-3637)  Internship in gerontopsychiatry - optional 2 (MED-3704)  Internship in gerontopsychiatry - optional 3 (MED-3705)  Internship in psychiatry - optional 2 (MED-3715)  Internship in forensic psychiatry - optional 2 (MED-3716)  Internship in forensic psychiatry - optional 3 (MED-3717)  Internship in forensic psychiatry - optional 2 (MED-3718)  Internship in forensic psychiatry - optional 3 (MED-3719)  Internship in subspecialized psychiatry - optional 2 (MED-3720)  Internship in subspecialized psychiatry - optional 3 (MED-3721)  Internship in personality disorders - optional 2 (MED-3785)  Internship in personality disorders - optional 3 (MED-3786)  Internship in child psychiatry - optional 2 (MED-3787)  Internship in child psychiatry - optional 3 (MED-3788)  Internship in psychiatric emergency (MED-3791)  Internship in diagnostic radiology (MED-3585)  Internship in diagnostic radiology outside the Laval University (MED-3586)  Internship in subspecialty diagnostic radiology (MED-3659)  Internship in subspecialty diagnostic radiology - optional 2 (MED-3722)  Internship in subspecialty diagnostic radiology - optional 3 (MED-3723)  Internship in radiation oncology (MED-3587)  Internship in radio-oncology outside network Université Laval (MED-3588)  Internship in radiation oncology - optional 3 (MED-3724)  Research internship (MED-3590)  Research internship - optional 2 (MED-3783)  Research internship - optional 3 (MED-3784)  IFMSA internship in other disciplines (MED-3643) | **Core**  **Elective** |
| ME008 | 1 document | **Core**  Molecules to Global Health (INDS 111)  Respiration (INDS 112)  Circulation (INDS 113)  Digestion and Metabolism (INDS 114)  Renal (INDS 115)  Defense (INDS 116)  Infection (INDS 117)  Movement (INDS 118)  Research Fundamentals 1 (INDS 123J1)  Research Fundamentals 1 (INDS 123J2)  Research Fundamentals 1 (INDS 123J3)  Longitudinal Family Medicine Experience (INDS 124J1)  Longitudinal Family Medicine Experience (INDS 124J2)  Longitudinal Family Medicine Experience (INDS 124J3)  Reflection and Evaluation 1(INDS 125J1)  Reflection and Evaluation 1 (INDS 125J2)  Reflection and Evaluation 1 (INDS 125J3)  Clinical Method 1 (INDS 119J1)  Clinical Method 1 (INDS 119J2)  Clinical Method 1 (INDS 119J3)  Physician Apprenticeship 1 (INDS 122J1)  Physician Apprenticeship 1 (INDS 122J2)  Physician Apprenticeship 1 (INDS 122J3)  **Elective** – none offered | **Core**  Reproduction and Sexuality (INDS 211)  Human Behaviour (INDS 212)  Research Fundamentals 2 (1 INDS 223)  Reflection and Evaluation 2 (INDS 225)  Community Health Alliance Project - C.H.A.P (INDS 224J1)  Community Health Alliance Project - C.H.A.P (INDS 224J2)  Community Health Alliance Project - C.H.A.P (INDS 224J3)  Clinical Method 2 (INDS 219)  Physician Apprenticeship 2 (INDS 222J1)  Physician Apprenticeship 2 (INDS 222J2)  Physician Apprenticeship 2 (INDS 222J3)  **Elective**  Global and Public Health Pre-Clerkship Elective (ELEC 200) | **Core**  TCP Anesthesia (ANAE 301)  TCP Family Medicine (FMED 301)  TCP Internal Medicine (IMED 301)  Transition to Clerkship (INDS 305)  TCP Integrated Assessment (INDS 323)  TCP Neurology (NEUR 301)  TCP Ophthalmology (OPTH 300)  TCP Pediatrics (PAED 301)  TCP Radiology (RADD 301)  TCP Surgery (SURG 301)  Mindful Medical Practice - Transition to Clinical Practice (1 INDS 300)  Medical Ethics and Health Law (INDS 302)  Formation of the Professional and Healer (INDS 320J1)  Formation of the Professional and Healer (INDS 320J2)  Formation of the Professional and Healer (INDS 320J3)  Physician Apprenticeship 3 (0 INDS 322J1)  Physician Apprenticeship 3 (INDS 322J2)  Physician Apprenticeship 3 (INDS 322J3)  **Elective**  Elective (ELEC 300) | **Core**  Family Medicine Clerkship (FMED 405)  Internal Medicine Clerkship (IMED 401)  Geriatric Medicine Clerkship (IMED 407)  Emergency Medicine Clerkship (INDS 408)  Transition to Residency (INDS 421)  Clerkship Integrated Assessment 1 (INDS 423)  Clerkship Integrated Assessment 2 (INDS 424)  Putting It All Together: Basic Science, Medicine and Society (INDS 426)  Public Health and Preventive Medicine Clerkship (INDS 427)  Obstetrics and Gynecology Clerkship (OBGY 401)  Pediatrics Clerkship (PAED 401)  Psychiatry Clerkship (PSYT 401)  Surgery Clerkship (SURG 402)  Physician Apprenticeship 4 (INDS 422D1)  Physician Apprenticeship 4 (INDS 422D2)  Roles in Interprofessional Teams (IPEA 500)  Communication in Interprofessional Teams (IPEA 501)  Patient-Centred Care in Action (IPEA 502)  **Elective**  Elective 1 Clerkship (ELEC 400)  Elective 2 Clerkship (ELEC 401)  Elective 3 Clerkship (ELEC 402)  Elective 4 Clerkship (ELEC 403)  Elective 5 Clerkship (ELEC 404) |
| ME009 Prep Year (5-year program in total) | | **Core**  Methodology in Medicine (MMD 1040)  Macroscopic Medicine (MMD 1041)  Molecular and Microscopic Medicine (MMD 1042)  Metabolic and Physiological Medicine (MMD 1043)  Relational Medicine 1 (MMD 10441)  Relational Medicine 2  Elective (MMD 10442)  Themes in Developmental Medicine (MMD 1045)  Professional Practice in Medicine (MMD 1046)  Topics in Clinical Medicine (MMD 1047)  Factual Medicine (MMD 1048)  **Elective**  Patient and Family Awareness (MMD10292) |  | | |
| ME009 | 1 document | **Core**  Pathology and Immunology – no documents  Growth, Development and Aging – no documents  Medical Microbiology and Infectious Diseases (MMD3525)  Hematology (MMD3511I)  Neurology (MMD3511L)  Physical Sciences – no documents  Movement – no documents  **Elective (Year 1 or 2)**  Patient and Family Awareness (MMD10291)  First Nations Health (MMD1098)  Individual clinical work (MMD1087)  Individual Research Work 1 (MMD1088A)  Research Initiation (MMD1091)  Intervention with disadvantaged populations (MMD1097)  Immersion in Global Health (MMD1141)  Transition space and mental health (MMD1143)  Tropical Medicine (MMD1095)  International Clinical Immersion (MMD1142) | **Core**  Heart and Circulation – no documents  Breathing and Oxygenation – no documents  Kidneys and Urology – no documents  Nutrition (MMD3522)  Endocrinology (MMD3511D)  Multisystem Problems – no documents  **Elective**  Introduction to Rehabilitation Medicine (MMD1082) | **Core**  Internship in Medicine (MMD 3701)  Surgery Internship (MMD3702)  **Core (Year 3 or 4)**  Internal Medicine (MMD3511J)  Pediatrics Internship (MMD3703)  Community Health (MMD3513A)  Social Medicine Internship (MMD3556)  Obstetrics and Gynecology Internship (MMD3704)  Psychiatry Internship (MMD3705)  Family Medicine Internship (MMD3707)  Geriatric Internship (MMD4507)  Anesthesia and Resuscitation Internship (MMD4509)  Opthalmology Internship (MMD4510)  Community Medicine Internship (MMD 4511)  Emergency Medicine Internship (MMD 4513)  **Elective (Year 3 or 4)**  Surgery Initiation - Surgical Anatomy (UQTR MED1015)  Anesthesiology (MMD3519)  Pain Clinic (MMD3519)  Medical Biochemistry (MMD3524)  Internship in Diagnostic Radiology (MMD4524)  Cardiac Surgery (MMD3512A)  Digestive Surgery (MMD3512O)  General Surgery (MMD3512B)  Pediatric General Surgery (MMD3512C)  Hepatobiliary Surgery (MMD3512P)  Orthopaedic Surgery (MMD3512D)  Plastic Surgery (MMD3512E)  Thoracic Surgery (MMD3512K)  Vascular Surgery (MMD3512L)  Neurosurgery (MMD3512H)  Ear, Nose and Throat (MMD3512I)  Surgical Intensive Care (MMD3512N)  Urology (MMD3512J)  Pediatric Urology (MMD3512J)  Family Medicine (MMD3517)  Acute Care Internship and Integration of Targeted Ultrasound with Family Medicine Practice in the Region  Addiction Medicine  Palliative Care (MMD3517E)  Pediatric Palliative Care (MMD3517E)  General Medicine (Psychiatry/Intellectual Disability) (MMD3517E)  Allergy and Clinical Immunology (MMD3511A)  Cardiology (MMD3511B)  Dermatology (MMD3511C)  Gastroenterology (MMD3511E)  Hematology and Oncology (MMD3511R)  Clinical Hepatology (MMD3511V)  Gene Medicine (MMD3511)  Short Hospitalization Unit (MMD3517)  Nephrology (MMD3511K)  Medical Oncology (MMD3511M)  Psychiatry (MMD3511N)  Pheumology (MMD3511O)  Rheumatology (MMD3511P)  Intensive Care (MMD3511W)  General Obstetrics (MMD3518B)  Gynecological Oncology (MMD3518C)  Gynecological Outpatient Care (MMD3518A)  Urogynecology (MMD3627A)  High-Risk Pregnancy (MMD3518A)  Fertility – Centre for Assisted Reproduction (MMD3518A)  Ophthalmology (MMD3518A)  Optometry and Oculovisual Care  Medical and Surgical Pathology (MMD3523)  Pediatric Allergy (MMD3515Y)  Pediatric Cardiology (MMD3515M)  General Pediatric Consultation and Hospitalization (MMD3515)  Pediatric Dermatology (MMD3515N)  Pediatric Endocrinology (MMD3515O)  Pediatric Gastroenterology (MMD3515P)  Genetics - Metabolic Diseases (MMD3515V)  Hematology – Pediatric Oncology (MMD3515Q)  Pediatric Rheumatology Immunology (MMD3515L)  Pediatric Infectious Diseases (MMD3515J)  Abuse/Socio-Legal (MMD3515)  Adolescent Medicine (MMD3515E)  Neonatology (MMD3515A)  Pediatric Nephrology (MMD3515R)  Outpatient Pediatrics (MMD3515K)  Developmental Pediatrics (MMD3515C)  International Pediatrics (MMD3515W)  Social Pediatrics (MMD3515X)  Pediatric Physiatry Rehabilitation (MMD3515T)  Pediatric Pulmonology (MMD3515U)  Pediatric Intensive Care (MMD3515I)  Pediatric Emergency (MD3515H)  Clinical Pharmacology (MMD3526)  Psychiatry (MMD3516)  Gerontopsychiatry (MMD3516C)  Psychosomatic Medicine (MMD3516D)  Child Psychiatry – Childhood & Adolescence (MMD3516A)  Adult Psychiatry – Psychiatric Emergency (MMD3516B)  Adult-Module Psychiatry of Relational Disorders (MMD3516B)  Adolescent Psychiatry (MMD3516A)  Forensic Psychiatry (MMD3516E)  Addition Psychiatry (MMD3516)  Psychogeriatrics (MMD3516C)  Outpatient Child Psychiatry (MMD3516A)  Psychiatry – Japanese Clinic (MMD3516)  Behavioral Psychiatry: Case Exposure to Palliative Care and Neurological Sciences (MMD3516)  Nuclear Medicine (MMD3520B)  Diagnostic Neuroradiology (MMD3520F)  Interventional Neurology (MMD3520D)  General Radiology (MMD3520A)  Musculoskeletal Radiology (MMD3520)  Digestive Radiology (MMD3520)  Thoracic Radiology (MMD3520)  Interventional Radiology (Angioradiology) (MMD3520E)  Oncology Radiology (MMD3520C)  Vascular Radiology (MMD3520)  Radiology Research (MMD3513B)  Radiology Oncology Research Oncology (MMD3513B)  Emergency Care (MMD3517B)  Cardiological Emergencies (MMD3517B)  Ultrasound/Emergency (MMD3517B) | **Core**  Palliative Care Internship (MMD 3557)  **Elective** |
| ME010 | 1 document | **Core**  Prevention in Public Health in Medical Practice (MSP171)  Strategic Medical Training I (MSP108)  Introduction to Leadership in Medicine (MSP109)  Foundations of Medical Research I (MSP105)  Medical Research Project I (MSP166)  Introduction to the medical profession (MSP110)  Frequent Situations in Women's Health (MSP112)  Frequent abdominal conditions (MSP 113)  Frequent situations of the upper limb (MSP114)  Frequent Mental Health Situations (MSP115)  Frequent situations in growth and development (MSP121)  Frequent pediatric situations (MSP122)  Frequent neurosensory situations (MSP123)  Frequent urogenital events (MSP125)  Frequent metabolic situations (MSP131)  Frequent systemic situations (MSP132)  Frequent situations of the lower limb or vascular (MSP134)  Assessment and management of frequent situations I (MSP145)  Service Learning in the Community - Integrative Activity in Medicine I (MSP146)  Meeting the Challenges of Professional Practice I (MSP161)  **Elective** – no documents/ indication if offered | **Core**  Promotion of health in medical practice (MSP271)  Effective presentation in medicine (MSP202)  Strategic Medical Education II (MSP208)  Project Leadership in Medicine (MSP280)  Foundations of Medical Research II (MSP205)  Medical Research Project II (MSP266)  Frequent or severe neurodevelopmental situations (MSP211)  Frequent or severe traumatic or axial situations (MSP212)  Frequent or severe situations in reproduction or sexuality (MSP213)  Frequent or severe situations in mental or social health (MSP214)  Frequent or severe - renal or electrolytic (MSP216)  Frequent or severe thoracic situations (MSP221)  Frequent or severe systemic and oncological conditions (MSP222  Frequent or severe systemic and abdominal conditions (MSP223)  Frequent or severe systemic and hematologic situations (MSP224)  Frequent complex neurosensory situations (MSP231)  Assessment and management of frequent or serious situations II (MSP245)  Service Learning in the Community - Integrative Medicine II (MSP246)  Meeting the Challenges of Professional Practice II (MSP261)  **Elective** – no documents | **Core**  Clinical Supervision and Learning (MSP302)  Strategic Medical Training III (MSP308)  Clinical Leadership in Medicine (MSP309)  Applied Research in Medicine (MSP305)  Frequent, complex and multi-systemic situations of young adults (MSP311)  Complex and multi-systemic frequent situations in adults (MSP312)  Complex end-of-life situations (MSP313)  Assessment and management of complex frequent situations III (MSP341)  Meeting the Challenges of Professional Practice III (MSP361)  Mandatory internship in anesthesia (MST111)  Mandatory internship in internal medicine (MST112)  Obligatory internship in surgery (MST113)  Compulsory internship in pediatrics (MST 114)  Mandatory internship in obstetrics and gynecology (MST115)  Mandatory internship in psychiatry (MST116)  Compulsory internship in family medicine / first line (MST117)  Mandatory internship in prevention and promotion of population health (MST118)  **Elective**  Elective internship I (MST101)  Elective internship 2A (MST102)  Elective internship 2B (MST103)  Elective placement 2C (MST104)  2D elective internship (MST105)  Elective internship 2E (MST106)  Elective internship 2F (MST107)  Cardiology internship (MST121)  Internship in dermatology (MST122)  Internship in Endocrinology (MST123)  Internship in gastroenterology (MST124)  Geriatric internship (MST125)  Internship in hemato-cytogenetics (MST126)  Internship in infectious diseases (MST127)  Internship in Nephrology (MST128)  Internship in Neurology (MST129)  Internship in pneumology (MST130)  Mandatory internship in specialized medicine in rheumatology (MST131)  Selective Stage I in Cardiology (MST141)  Selective internship I in dermatology (MST142)  Selective Stage I in Endocrinology (MST143)  Selective Stage I in Gastroenterology (MST144)  Selective Stage I in Geriatrics (MST145)  Selective Stage I in Hematology-Cytogenetics (MST146)  Selective Stage I in infectious diseases (MST147)  Selective Stage I in Nephrology (MST148)  Selective internship I in neurology (MST149)  Selective Stage I in Pneumology (MST150)  Selective Stage I in Rheumatology (MST151)  Selective internship I in medical intensive care (MST152)  Selective Stage I in Plastic Surgery (MST161)  Selective Stage I in Cardiac Surgery (MST162)  Selective Stage I in Cardiovascular and Thoracic Surgery (MST163)  Selective Stage I in Neurosurgery (MST164)  Selective Stage I in ophthalmology (MST165)  Selective Stage I in Otolaryngology (MST166)  Selective Stage I in Orthopedic Surgery (167)  Selective Stage I in Urology (MST168)  Selective Stage I in Surgical Intensive Care (MST169)  Selective Stage I in Mixed Intensive Care (MST170)  Pediatric / allergic-immuno selective course (MST181)  Selective Stage I in Cardiopediatrics (MST182)  Selective Stage I in Endocrinopediatrics (MST183)  Selective Stage I in gastropediatrics (MST184)  Selective Stage I in Hematopediatrics (MST185)  Selective Stage I in Neonatology (MST186)  Selective Stage I in Nephropediatrics (MST187)  Selective internship I in neuropediatrics (MST188)  Selective Stage I in pneumopediatrics (MST189)  Selective Stage I in Pediatric Critical Care (MST190)  Selective internship I in pediatrics of development (MST191)  Selective Stage I in Infectiopediatrics (MST192)  Integrated Stage A - selective II (MST201)  Integrated course B - selective II (MST202)  Selective Stage II in Cardiology (MST203)  Selective Stage II in Dermatology (MST204)  Selective Stage II in Endocrinology (MST205)  Selective Stage II in Gastroenterology (MST206)  Selective Stage II in Geriatrics (MST207)  Selective Stage II in Hematology-Cytogenetics (MST208)  Selective Stage II in infectious diseases (MST209)  Selective Stage II in Nephrology (MST210)  Selective II internship in neurology (MST211)  Selective Stage II in Pneumology (MST212)  Selective Stage II in Rheumatology (MST213)  Selective Stage II in Medical Critical Care (MST214)  Selective Stage II in Plastic Surgery (MST221)  Selective Stage II in Cardiac Surgery (MST222)  Selective Stage II in Cardiovascular and Thoracic Surgery (MST223)  Selective Stage II in Neurosurgery (MST224)  Selective Stage II in ophthalmology (MST225)  Selective Stage II in Otolaryngology (MST226)  Selective Stage II in Orthopedic Surgery (MST227)  Selective Stage II in Urology (MST228)  Selective Stage II in Surgical Intensive Care (MST229)  Selective Stage II in Mixed Intensive Care (MST230)  Selective Stage II in Pediatrics / Allergy-Immunology (MST241)  Selective II course in cardiopediatrics (MST242)  Selective II internship in endocrinopediatrics (MST243)  Selective Stage II in gastropediatrics (MST244)  Selective Stage II in Hematopediatrics (MST245)  Selective Stage II in Neonatology (MST246)  Selective Stage II in Nephropediatrics (MST247)  Selective Stage II in pneumopediatrics (MST249)  Selective Stage II in Pediatric Critical Care (MST250)  Selective Stage II in Pediatric Development (MST251)  Selective Stage II Infectiopediatrics (MST252)  Integrated Stage A - selective III (MST261)  Integrated Stage B - selective III (MST262)  Selective Stage III in Obstetrics and Gynecology (MST263)  Selective Stage III in Psychiatry (MST264) | **Core**  Strategic Medical Training IV (MSP408)  Assessment and management of situations requiring personalized support IV (MSP441)  Preparation for practice in residence (MSP442)  Elective Stage III (MST468)  Meeting the Challenges of Professional Practice IV (MSP461)  Mandatory internship in anesthesia (MST311)  Mandatory internship in internal medicine (MST312)  Obligatory internship in surgery (MST313)  Compulsory internship in pediatrics (MST 314)  Mandatory internship in obstetrics and gynecology (MST315)  Mandatory internship in psychiatry (MST316)  Compulsory internship in family medicine / first line (MST317)  Mandatory internship in prevention and promotion of population health (MST318)  Mandatory internship in cardiology (MST321)  Mandatory internship in dermatology (MST322)  Mandatory internship in endocrinology (MST323)  Mandatory internship in gastroenterology (MST324)  Mandatory internship in geriatrics (MST325)  Mandatory internship in hematology-cytogenetics (MST326)  Mandatory internship in infectious diseases (MST327)  Mandatory internship in nephrology (MST328)  Mandatory internship in neurology (MST329)  Mandatory internship in pneumology (MST330)  Mandatory internship in rheumatology (MST331)  **Elective**  Selective Stage I in Cardiology (MST341)  Selective internship I in dermatology (MST342)  Selective Stage I in Endocrinology (MST343)  Selective Stage I in Gastroenterology (MST344)  Selective Stage I in Geriatrics (MST345)  Selective Stage I in Hematology-Cytogenetics (MST346)  Selective Stage I in infectious diseases (MST347)  Selective Stage I in Nephrology (MST348)  Selective internship I in neurology (MST349)  Selective Stage I in Pneumology (MST350)  Selective Stage I in Rheumatology (MST351)  Selective internship I in medical intensive care (MST352)  Selective Stage I in Plastic Surgery (MST361)  Selective Stage I in Cardiac Surgery (MST362)  Selective Stage I in Cardiovascular and Thoracic Surgery (MST363)  Selective Stage I in Neurosurgery (MST364)  Selective Stage I in ophthalmology (MST365)  Selective Stage I in Otolaryngology (MST366)  Selective Stage I in Orthopedic Surgery (367)  Selective Stage I in Urology (MST368)  Selective Stage I in Surgical Intensive Care (MST369)  Selective Stage I in Mixed Intensive Care (MST370)  Pediatric / allergic-immuno selective course (MST381)  Selective Stage I in Cardiopediatrics (MST382)  Selective Stage I in Endocrinopediatrics (MST383)  Selective Stage I in gastropediatrics (MST384)  Selective Stage I in Hematopediatrics (MST385)  Selective Stage I in Neonatology (MST386)  Selective Stage I in Nephropediatrics (MST387)  Selective internship I in neuropediatrics (MST388)  Selective Stage I in pneumopediatrics (MST389)  Selective Stage I in Pediatric Critical Care (MST390)  Selective internship I in pediatrics of development (MST391)  Selective Stage I in Infectiopediatrics (MST392)  Integrated Stage A - selective II (MST401)  Integrated course B - selective II (MST402)  Selective Stage II in Cardiology (MST403)  Selective Stage II in Dermatology (MST404)  Selective Stage II in Endocrinology (MST405)  Selective Stage II in Gastroenterology (MST406)  Selective Stage II in Geriatrics (MST407)  Selective Stage II in Hematology-Cytogenetics (MST408)  Selective Stage II in infectious diseases (MST409)  Selective Stage II in Nephrology (MST410)  Selective II internship in neurology (MST411)  Selective Stage II in Pneumology (MST412)  Selective Stage II in Rheumatology (MST413)  Selective Stage II in Medical Critical Care (MST414)  Selective Stage II in Plastic Surgery (MST421)  Selective Stage II in Cardiac Surgery (MST422)  Selective Stage II in Cardiovascular and Thoracic Surgery (MST423)  Selective Stage II in Neurosurgery (MST424)  Selective Stage II in ophthalmology (MST425)  Selective Stage II in Otolaryngology (MST426)  Selective Stage II in Orthopedic Surgery (MST427)  Selective Stage II in Urology (MST428)  Selective Stage II in Surgical Intensive Care (MST429)  Selective Stage II in Mixed Intensive Care (MST430)  Selective Stage II in Pediatrics / Allergy-Immunology (MST441)  Selective II course in cardiopediatrics (MST442)  Selective II internship in endocrinopediatrics (MST443)  Selective Stage II in gastropediatrics (MST444)  Selective Stage II in Hematopediatrics (MST445)  Selective Stage II in Neonatology (MST446)  Selective Stage II in Nephropediatrics (MST447)  Selective Stage II in pneumopediatrics (MST449)  Selective Stage II in Pediatric Critical Care (MST450)  Selective Stage II in Pediatric Development (MST451)  Selective Stage II Infectiopediatrics (MST452)  Integrated Stage A - selective III (MST461)  Integrated Stage B - selective III (MST462)  Selective Stage III in Obstetrics and Gynecology (MST463)  Selective Stage III in Psychiatry (MST464)  Professional Development Project in Medicine - Population Health Promotion (MSP501)  Professional Development Project in Medicine – Training (MSP502)  Professional Development Project in Medicine – Management (MSP503)  Professional Development Project in Medicine – Research (MSP505)  Professional Development Project in Medicine – Care (MSP507)  Professional Development Project in Medicine - Internship in Prevention and Promotion of Global Health (MSP511)  Professional Development Project in Medicine - Applied Research Internship (MSP515)  Professional Development Project in Medicine - Elective V (MSP521)  Professional Development Project in Medicine - Remediation Activities (MSP522)  Specific Project for Professional Development in Medicine I (MSP523)  Specific Project for Professional Development in Medicine II (MSP524)  Professional Development Project in Medicine - Service Learning in the Community (MSP542) |
| ME011 | 1 document | **Core**  Basic Life Support for Health Care Providers (MEDC 101.0)  Success in Medical School I (MEDC 111.0)  Medicine and Society I (MEDC 112.3)  Clinical Skills I (MEDC 113.8)  Clinical Integration I (MEDC 114.4)  Principles of Medical Science (MEDC 115.18)  Medicine and Society II (MEDC 122.3)  Clinical Skills II (MEDC 123.8)  Clinical Integration II (MEDC 124.4)  Foundations of Clinical Medicine I (MEDC 126.18)  **Elective**  Extra Curricular Medical Experience I (MEDC 100.0) | **Core**  Success in Medical School II (MEDC 211.0)  Medicine and Society III (MEDC 212.3)  Clinical Skills III (MEDC 213.8)  Clinical Integration III (MEDC 214.4)  Foundations of Clinical Medicine II (MEDC 216.18)  Medicine and Society IV (MEDC 222.3)  Clinical Skills IV (MEDC 223.8)  Clinical Integration IV (MEDC 224.4)  Foundations of Clinical Medicine III (MEDC 226.18)  **Elective**  Extra Curricular Medical Experience II (MEDC 200.0) | **Core**  Core Clinical Rotations (MEDC 307.50)  Saskatchewan Longitudinal Integrated Clerkship SLIC (MEDC 306.50)  Selected Topics in Medicine (MEDC 308.16)  Selective Clinical Rotations (MEDC 309.8)  Success in Medical School Clerkship III (MEDC 311.0)  **Elective** | **Core**  Elective Clinical Rotations (MEDC 407.32)  Preparation for Residency (MEDC 409.8)  **Elective**  Clerkship Extension Course (MEDC 505.15)  Aboriginal Health – no documents  Anesthesia - no documents  CaRMS Interviews - no documents  Clinical Ultrasound  Community Health, Epidemiology & Quality Improvement - no documents  Emergency Medicine - no documents  External Elective - no documents  Family Medicine - no documents  Internal Medicine - no documents  Medical Imaging - no documents  OSCE - no documents  Obstetrics & Gynecology - no documents  Occupational Health - no documents  Ophthalmology - no documents  Palliative Care - no documents  Pathology - no documents  Pediatrics - no documents  Physical Medicine & Rehabilitation - no documents  Psychiatry - no documents  Research - no documents  Surgery - no documents |
| ME012 | 1 document | **Core**  Foundations of Medical Practice (MEDD 411) [+1 additional file provided by school (A)], [+1 additional file provided by school (B)], [+1 additional file provided by school (C)]  Foundations of Medical Practice II (MEDD 412) [+1 additional file provided by school (A)], [+1 additional file provided by school (B)], [+1 additional file provided by school (C)]  Foundations of Scholarship and Flexible Enhanced Learning (MEDD 419)  **Elective** – none offered | **Core**  Foundations of Medical Practice III (MEDD 421) [+1 additional file provided by school (B)], [+1 additional file provided by school (C)]  Transition into Clinical Education (MEDD 422) [+1 additional file provided by school (A)], [+1 additional file provided by school (B)]  Flexible Enhanced Learning II (MEDD 429)  **Elective**  - none offered | **Core**  Clerkship (MEDD 431) [+1 additional file provided by school (B)], [+1 additional file provided by school (C)]  Pediatrics  Obstetrics/Gynecology  Surgery - no documents  Orthopedics - no documents  Anesthesiology - no documents  Internal Medicine - no documents  Psychiatry  Emergency Medicine  Family Practice  Ambulatory Care - no documents  **Elective** – not clear if offered, no documents found | **Core**  Transition into Postgraduate Education and Practice (MEDD 448) [+1 additional file provided by school (A)], [+1 additional file provided by school (C)]  Flexible Enhanced Learning III (MEDD 449)  **Elective**  MEDD 441 - no documents  MEDD 442 - no documents  MEDD 443 - no documents  MEDD 444 - no documents  MEDD 445 - no documents  MEDD 446 - no documents |
| ME013 | 1 document | **Core (Year 1 or 2)**  Foundation of Medicine (FM0)  Human Biology & Health (M1)  Health and Disease (M2)  Blood and Immunology (BI1)  Cardiovascular (CV1)  Respiratory (RS1)  Neurosciences (NS1)  Musculoskeletal (MSK)  Endocrine and Metabolism (EM)  Women’s Reproductive Health (WRH)  Gastroenterology, Hepatology, and Nutrition (GHN)  Urinary Tract (UT)  Infectious Diseases and Therapeutics (IDT)  Introduction to Oncology (ONC)  Clinical Reasoning (CR)  Clinical Skills (CS)  Professionalism (PF)  Population Health (PH)  Indigenous Health (IH)  Consolidation Module 3 (CM)  **Elective** – none offered | **Core**  **Elective** | **Core**  Transition to Clerkship (M4)  Core Clinical Rotations and UGME Academic Half Days (M5)  Surgery - no documents  Anesthesia - no documents  Pediatrics - no documents  Obstetrics & Gynecology - no documents  Internal Medicine - no documents  Emergency Medicine - no documents  Psychiatry - no documents  Family Medicine/Public Health - no documents  **Elective** – none offered | **Core**  Electives and CaRMS (M6)  Transition to Residency (M7)  **Elective**  Anesthesiology  Aboriginal Health  Indigenous Health  Public Health/Preventive Medicine (Regional)  Public Health/Preventive Medicine (FNIHB)  Occupational Health  Manitoba Centre for Health Policy  Public Health/Preventive Medicine (Provincial)  Emergency Medicine  Family Medicine (Rural or Urban)  Health Care Ethics  Cardiology  Clinical Immunology & Adult Allergy  Clinical Teaching Unit – no document  Dermatology  Endocrinology  Gastroenterology  Geriatric Medicine  Hematology  Hepatology  ICU/Critical Care  Infectious Diseases  Nephrology  Neurology  Oncology  Palliative Care  Physical Medicine & Rehab  Respiratory  Rheumatolgy  Medical Genetics  Ongomiizwin Health Services (OHS) – Churchill  Ongomiizwin Health Services (OHS) - Fly-In  Ongomiizwin Health Services (OHS)  - Norway House  Gynecology  Gynecologic Oncology  High Risk/Maternal Fetal Medicine  Obstetrics  Ophthalmology  Otolaryngology  Pathology  Allergy  Cardiology  Child Development  Child Protection  Endocrinology  Gastroenterology  Hematology/Oncology  Infectious Diseases  Intensive Care Unit (PICU)  Neonatology  Nephrology  Palliative Care  Respiratory  Rheumatology  Neurology Pediatric  Anxiety/Mood Disorders – no document  Child & Adolescent  CODI  Community  Consultation Liaison  Eating Disorders – no document  Forensics  General (HSC)  General (SBGH)  Intensive Care Unit  Psychotherapy  Radiation Oncology  Diagnostic Radiology  Cardiac Surgery  Head & Neck Surgical Oncology Service  Neurosurgery  Pediatric Orthopedic Surgery  Reconstructive Orthopedic Surgery  Spine Orthopedic Surgery  Sports Orthopedic Surgery  Trauma Orthopedic Surgery  Plastics  Urology  General Surgery A Service  General Surgery B Service  General Surgery ACSS  General Surgery Gold Service  General Surgery Green Service  General Surgery Orange Service  General Pediatric Surgery  Thoracic  Surgical Intensive Care Unit  Vascular Surgery |
| ME014 | 1 document [+1 additional file provided by school] | **Core**  The Healthy Person (MED 5710)  Clinical Skills I (MED 5720)  Physician Competencies I (MED 5730)  Community Engagement I (MED 5740)  **Core (Year 1 or 2)**  The Patient: Acute or Episodic Health Problems (MED 6750)  Clinical Skills II (MED 6770)  Physician Competencies II (MED 6770)  Community Engagement II (MED 6780)  **Elective** – none offered | **Core**  The Patient: Chronic Conditions (MED 7710)  Clinical Skills III (MED 7720)  Physician Competencies III (MED 7730)  Phase 4 Preparation (MED 7740)  Community Engagement III (MED 7750)  **Elective** – none offered | **Core (Year 3 or 4)**  Internal Medicine Rotation  Obstetrics and Gynecology Rotation  Pediatrics Rotation  Psychiatry Rotation  Rural Family Medicine Rotation  Surgery Rotation  Advanced Procedural Competencies (MED 8720)  Practice Continuum (MED 8750)  **Elective (Year 3 or 4)**  Advanced Practice Integration (MED 8740)  Electives (MED 8730) | **Core**  **Elective** |
| ME015 | 1 document | **Core**  Foundations of Medicine  Host Defense  Metabolism and Homeostasis  Human Development  Professional Competencies  Clinical Skills I - no documents  Rural Week  Skilled Clinician Program  Research in Medicine (RIM)  Interprofessional Health Education  Volunteer Patient Program  **Elective**  Electives – Med 1 | **Core**  Foundations of Medicine II  Neurosciences  Metabolism II  Musculoskeletal and Dermatology  Integration  Clinical Skills II - no documents  Professional Competencies II  Skilled Clinician Program  Research in Medicine (RIM)  Interprofessional Health Education  **Elective**  Electives – Med 2 | **Core**  Introduction to Clerkship  PIER 1  PIER 2  **Core (Year 3 or 4)**  Emergency Medicine Clerkship Unit  Family Medicine Clerkship Unit  Internal Medicine Clerkship Unit  Obstetrics and Gynecology Clerkship Unit  Pediatric Clerkship Unit  Psychiatry Clerkship Unit  Surgery Clerkship Unit  Care of the Elderly Clerkship Unit  **Elective (Year 3 or 4)**  Electives Clerkship Unit | **Core**  CRAM Clerkship Unit  **Elective** |
| ME016 | 1 document | **Core**  Northern and Rural Health (MEDS 5005)  Personal and Professional Aspects of Medical Practice (MEDS 5025)  Social and Population Health (MEDS 5045)  Foundations of Medicine (MEDS 5065)  Clinical Skills in Health Care (MEDS 5085)  Indigenous Community Placement  **Elective** – none offered | **Core**  Northern and Rural Health (MEDS 5205)  Personal and Professional Aspects of Medical Practice (MEDS 5225)  Social and Population Health (MEDS 5245)  Foundations of Medicine (MEDS 5265)  Clinical Skills in Health Care (MEDS 5285)  Rural/Remote Community Placement  **Elective**  Elective (MEDS 5305) | **Core**  Northern and Rural Health (MEDS 5405)  Personal and Professional Aspects of Medical Practice (MEDS 5425)  Social and Population Health (MEDS 5445)  Foundations of Medicine (MEDS 5465)  Clinical Skills in Health Care (MEDS 5485)  Comprehensive Community Clerkship (CCC)  **Elective** – not clear if any are offered, no documents | **Core**  Northern and Rural Health (MEDS 5605)  Personal and Professional Aspects of Medical Practice (MEDS 5625)  Social and Population Health (MEDS 5645)  Foundations of Medicine (MEDS 5665)  Clinical Skills in Health Care (MEDS 5685)  Internal Medicine (MEDS 5710)  Surgery (MEDS 5711)  Women’s Health (MEDS 5713)  Children’s Health (MEDS 5714)  Mental Health (MEDS 5715)  Emergency Medicine (MEDS 5716)  The Large Urban Health Science Centre Locations  **Elective**  Elective/Medical/Specialty (MEDS 5750) - no documents  Elective/Surgical/Specialty (MEDS 5751) - no documents  Elective/Women's Health /Specialty (MEDS 5752) - no documents  Elective/Children's Health/Specialty (MEDS 5753) - no documents  Elective/Internal Medicine/Specialty (MEDS 5754) - no documents  Elective/Mental Health Medicine/Specialty (MEDS 5755) - no documents  Elective/Family Medicine/Specialty (MEDS 5756) - no documents  Elective/Research (MEDS 5757) - no documents  Elective/Human Sciences (MEDS 5758) - no documents  Elective/Laboratory Medicine/Pathology/Specialty (MEDS 5759) - no documents  Elective/Diagnostic Imaging/Nuclear Medicine/Specialty (MEDS 5770) - no documents  Elective/Emergency Medicine (MEDS 5771) - no documents  Elective/Community Medicine (MEDS 5772) - no documents  Elective/Anesthesia (MEDS 5773) - no documents |
