# Supplementary material for: Medical student exposure to women’s health concepts and practices: a content analysis of curriculum at Canadian medical schools
Source: BMC Med Educ. 2021 Aug 18;21:435. doi: 10.1186/s12909-021-02873-8 (PMC8371837; doi:10.1186/s12909-021-02873-8)
Supplement: Supplementary file 2 — Additional file 2: Guidance for extracting WH data. Definitions and guidance for WH data extraction. Table listing WH domains, descriptions, extraction criteria, and brief and more detailed examples extracted from documents to illustrate domains [file 12909_2021_2873_MOESM2_ESM.docx]

**Medical student exposure to women’s health concepts and practices: A content analysis of curriculum at Canadian medical schools**

Natalie N. Anderson, MPH, Anna R. Gagliardi, PhD, Toronto General Hospital Research Institute, University Health Network, Toronto, Canada

**Additional File 2. Definitions and guidance for WH data extraction**

| Domain | Description | Extraction Criteria | Examples from included documents | |
| --- | --- | --- | --- | --- |
|  |  |  | Brief | Detailed |
| Gender | Refers to the socially constructed roles, behaviours, identities and expressions of women, men and gender diverse people. Gender roles, norms and relations influence people’s susceptibility to different health conditions and diseases, affecting their mental health, physical health and wellbeing. | Included any mention of socially constructed roles and behaviours, sex and gender based medicine, gender identities, gender expression, or gender-related factors. | Topics addressed will include: Gender and health (ME005) | Develop collaborative and respectful relationships that demonstrate gender, cultural and age related awareness and respect. (ME003)  Formulate a supportive management plan that is sensitive to gender issues (ME004) |
| Women’s health | Refers to the branch of medicine that focuses on the diagnosis and treatment of diseases and conditions that affect women’s emotional and physical well-being. | Information regarding learning the knowledge, skills and attitudes relevant to women’s health and healthcare for women. | This course will highlight integrated Clinical Presentations related to Women’s Health and Pediatrics. (ME001) | Clinical sciences applicable to situations in women's health: main signs and symptoms, habits of life, paraclinical indices relevant to the differential diagnosis, risk factors and current measures of screening and preventive interventions, lifestyle and stages of change, safety of the patient and general principles of the treatments as well as the basic principles of their judicious use, their indications, contraindications and side effects related to the frequent clinical entities in the young adult. (ME011) |
| Determinants of health and access to health services | A broad range of social, personal, economic and environmental factors that influence individual and population health: income and social status, employment and working conditions, ethnicity or culture, education and literacy, childhood experiences, physical environments, social supports and coping skills, healthy behaviours, access to health services, or biology and genetic endowment. | Any reference to acknowledging, understanding or identifying patient’s determinants of health, as well as implications and actions taken from such recognition. | Apply the social determinants of health in the context of the population served by the agency with whom you're working with. (ME003) | The competent graduate recognizes the diverse factors that influence the health of the individual and the community; identifies the sociocultural, familial, psychological, economic, environmental, legal, political and spiritual factors impacting health care and health care delivery; and responds to these factors by planning and advocating the appropriate course of action at both the individual and the  community level. (ME005)  The student recognizes the influence of social determinants on the ability of his or her patients to influence their health status and proposes solutions to take into account takes into account the adverse effects of certain determinants (ME010)  Know the determinants of health and their impacts, interpret data on determinants and health status, recognize its role as a physician and know the role of the partners involved in promoting the health of populations, know the main strategies for promoting health and justify the choice based on context, based on the interpretation of evidence and considering vulnerable populations, understand the ethical principles and benchmarks that guide health promotion activities. (ME011) |
| Frameworks or models of women’s health or patient-centred care for women | PCC is an approach to care that adopts patient and family needs and decisions within healthcare trajectories, and many frameworks and models exist pertaining to this concept. | Theoretical or conceptual frameworks, or models describing the components of women’s health or what optimizes care for women | Using standardized patients, students will have the opportunity to develop skills in communicating with patients of varying cultural backgrounds and life cycle stages, and interacting with patients following a patient-centered model of care. (ME017) | Accounting for the culture, context, experience, feelings, and expectations of the whole person under the patient-centered care model, with a focus on geriatric medicine (care of the elderly) and oncology (cancers), Integration explores the relationship of the physician to the patient and patient-family through ethical, social, and political lenses (ME016) |
| Considerations when delivering or supporting the care of women for any health issue | Any other principles, recommendations, or guidance pertaining to care of women | How to organize, structure, deliver or support care for women | Perform a physical and cognitive assessment without causing the patient embarrassment. (ME004) | Demonstrate a safe and comfortable communication style and sensitive approach throughout the patient encounter, using sensitive and appropriate language, history taking skills, eye contact, gestures, body language, privacy, draping techniques and providing sensitive feedback and ongoing explanations as the exam proceeds (ME013) |
